# Probing the nucleotide-binding activity of a redox sensor: two-component regulatory control in chloroplasts

**DOI:** 10.1007/s11120-016-0229-y

**Published:** 2016-02-12

**Authors:** Iskander M. Ibrahim, Sujith Puthiyaveetil, Christine Khan, John F. Allen

**Affiliations:** 1 Faculty of Engineering and Science, University of Greenwich, Chatham Maritime, Kent, UK; 2 Institute of Biological Chemistry, Washington State University, Pullman, WA USA; 3 12 Ballamaddrell, Port Erin, Isle of Man UK; 4 Research Department of Genetics, Evolution and Environment, University College London, Darwin Building, Gower Street, London, WC1E 6BT UK

**Keywords:** Two-component regulatory system, Sensor histidine kinase, Response regulator, Chloroplast sensor kinase, Plastoquinone, Redox regulation, Chloroplast DNA, Transcription, Cyanobacteria, Photosystem stoichiometry

## Abstract

Two-component signal transduction systems mediate adaptation to environmental changes in bacteria, plants, fungi, and protists. Each two-component system consists of a sensor histidine kinase and a response regulator. Chloroplast sensor kinase (CSK) is a modified sensor histidine kinase found in chloroplasts—photosynthetic organelles of plants and algae. CSK regulates the transcription of chloroplast genes in response to changes in photosynthetic electron transport. In this study, the full-length and truncated forms of *Arabidopsis* CSK proteins were overexpressed and purified in order to characterise their kinase and redox sensing activities. Our results show that CSK contains a modified kinase catalytic domain that binds ATP with high affinity and forms a quinone adduct that may confer redox sensing activity.

## Introduction

Photosynthesis converts light energy from the sun into useful chemical energy. This important biological process takes place in some prokaryotes and in chloroplasts—bioenergetic organelles of eukaryotic plants and algae. In photosynthesis, in chloroplasts and cyanobacteria, light-driven primary electron transfer is carried out by the photochemical reaction centres of two photosystems, photosystem II (PS II) and photosystem I (PS I). A mobile electron carrier—plastoquinone (PQ)—in the thylakoid membrane is a link in the electron transport chain that connects these two reaction centres in series. The reduction–oxidation (redox) state of the pool of PQ molecules determines distribution of excitation energy between PS II and PS I by controlling the reversible phosphorylation of polypeptides of light-harvesting complex II (LHC II) in chloroplasts (Allen 1992; Allen et al. 1981). The redox state of the PQ pool also controls transcription of chloroplast DNA, regulating expression of genes that encode reaction-centre proteins of PS II and PS I, thus initiating a long-term light quality acclimatory process known as photosystem stoichiometry adjustment (Pfannschmidt et al. 1999). In cyanobacteria, prokaryotes from which chloroplasts originated, a similar redox control of photosystem stoichiometry is observed (Fujita 1997; Murakami et al. 1997).

Redox chemistry in the thylakoid membrane is coupled to chloroplast DNA transcription by a bacterial-type two-component signal transduction system (TCS). TCSs are the predominant signalling mechanisms in prokaryotic organisms. TCSs consist of two proteins, a sensor histidine kinase (component 1) and a response regulator (component 2) (Stock et al. 2000). Chloroplasts contain a modified bacterial-type sensor histidine kinase, named chloroplast sensor kinase (CSK) (Puthiyaveetil et al. 2008). In *Arabidopsis*, CSK is encoded by the nuclear gene *At1g67840* and imported into chloroplasts as a precursor protein synthesised in the cytosol. Under changing light quality conditions that perturb the redox state of the PQ pool, *Arabidopsis* plants lacking the *CSK* gene are unable to regulate transcription of the chloroplast reaction-centre gene *psaA* (encoding the PsaA protein of photosystem I), and are impaired in photosystem stoichiometry adjustments (Puthiyaveetil et al. 2008). CSK is therefore suggested as the sensor component in the signal transduction chain that underlies photosystem stoichiometry adjustments (Puthiyaveetil et al. 2008). The mechanisms by which the photosynthetic electron transport chain controls the activity of CSK, and how CSK regulates the transcription of the *psaA* gene, are under investigation (Puthiyaveetil et al. 2013). Here, we report on the ATP-binding activity of CSK using the overexpressed, purified full-length recombinant CSK protein. We show that the CSK has a modified ATP-binding site and yet it binds ATP with a *K*_d_ value similar to that of unmodified histidine kinases.

## Materials and methods

### Secondary structure prediction

The secondary structure of CSK was predicted using the PSIPRED programme (http://bioinf.cs.ucl.ac.uk/psipred/).

### Construction of recombinant plasmids

Coding sequences for the full-length *Arabidopsis* CSK protein (CSK_F) and for a truncated version (CSK_T) (amino acids 301 to 611) were amplified from a *CSK* cDNA clone using primer pairs listed in Table 1. The PCR fragment for CSK_F was digested with *NdeI* and *BamHI* endonucleases (New England BioLabs) and cloned into a customised pJC20 expression vector (ATCC). The PCR fragment for CSK_T was cloned into a Gateway pENTR entry vector (Invitrogen) and then mobilised into a customised pETG-10A destination expression vector (EMBL).

**Table 1:** Primers used for cloning full-length CSK (CSK_F) and truncated CSK (CSK_T). Sequences in lower case are restriction site overhangs

CSK-F	
Forward	GCCGTGcatatgCTTCTTT CTGCAATCGCTTC
Reverse	CGAggatccCTATGCTTCATTGGCTTC
CSK-T	
Forward	CACCATGCAGTCATCTTGGCAAAAC
Reverse	CTATGCTTCATTGGCTTC

### Expression and purification of recombinant CSK protein

Recombinant plasmids were transformed into BL21(DE3) chemically competent cells (stratagene). Transformed bacterial colonies grown on agar plates were used to inoculate starter cultures (20 ml each) in Luria broth (LB) growth medium (8) supplemented with 100 *μ*g mL^−1^ ampicillin as the selection agent. Each culture was grown overnight, then diluted 1:100 in 2 L LB media and grown at 37 °C to an optical density of ~0.55 at 600 nm before inducing protein expression by addition of isopropyl *β*-D-1-thiogalactopyranoside (IPTG) (Melford) to a final concentration of 100 µm. Bacterial cultures were grown for a further 16 h at 17 °C. Cells were harvested by centrifugation at 6000 rpm for 10 min. The pellet was re-suspended in 20 mL of resuspension buffer (300 mM NaCl, 50 mm NaH_2_PO_4_ pH 7.4, 25 mm imidazole, and 1 mm PMSF) and the cells lysed with an EmulsiFlex-C3 homogenizer (Avestin). Lysate was separated by centrifugation at 18,000 rpm for 20 min. The supernatant was applied to a Ni^2+^ affinity chromatography column (GE Healthcare) and the N-terminally poly-histidine tagged CSK protein was purified according to the column manufacturer’s instructions. For ATP-binding assay, the elution buffer in the purified protein was exchanged with ATP-binding buffer (10 mm NaCl and 10 mm tris–HCl (pH 8)) using the PD-10 desalting column (Amersham Biosciences).

### *In vitro* autophosphorylation assay in the presence of redox agents

Recombinant CSK protein at 2.5 µm concentration was mixed with the kinase reaction buffer (50 mm tris–HCl (pH 7.5), 50 mm KCl, 10 % glycerol, and 10 mm MgCl_2_) and the following redox agents: 2 mm K_3_Fe(CN)_6_, 6 mm DTT, 0.5 mm benzoquinone, and 0.5 mm hydroquinone in a final volume of 25 µL and incubated at room temperature for 30 min. Autophosphorylation was initiated by the addition of 5 µL of 5-fold concentrated ATP solution giving a final concentration of 0.5 mm ATP and a specific activity of 5 *µ*Ci [γ-^32^P]ATP (6000 Ci mmol^−1^) (Perkin-Elmer). Reactions proceeded for 60 min at 30 °C. The autophosphorylation reaction was stopped by addition of 6 µl of 5X Laemmli sample buffer (Laemmli 1970). Proteins were resolved on a 12 % SDS-PAGE gel, blotted onto a PVDF membrane and the ^32^P-labeling analysed by a phosphor screen.

### TNP-ATP-binding assay

TNP-ATP-binding assay was carried out in a total volume of 3 mL ATP-binding buffer containing 2 µm of CSK_T and 1 µm of TNP-ATP. Samples were prepared in a 1 cm X 4 cm quartz cuvette. Fluorescence measurement was carried out using Perkin-Elmer LS55 spectrofluorometer with the excitation wavelength set at 410 nm and the emission wavelength at 500–650 nm. Excitation and emission monochromators were at 5 nm and 10 nm bandwidth, respectively. TNP-ATP was excited at 410 nm and the fluorescence emission was scanned in a wavelength range of 500–650 nm.

The TNP-ATP-binding titration was carried out by successive addition of varying concentration of TNP-ATP to 2 µm CSK_T diluted in ATP-binding buffer. Control titration without protein was performed in the same way. The fluorescence intensity increase at 550 nm was recorded. Subtracting the control fluorescence value from the sample value corrected for the buffer-TNP-ATP fluorescence. The total volume of TNP-ATP was less than 0.001 % of the total sample volume.

TNP-ATP displacement was carried out by successive addition of varying concentrations of ATP to a sample containing 2 µm CSK_T, 1 µm TNP-ATP in the ATP-binding buffer. Decrease in fluorescence emission intensity at 550 nm was monitored. The total volume of ATP added was less than 0.05 % of total sample volume.

Data were analysed as follows: the observed changes in fluorescence at any given concentrations of TNP-ATP (Δ*F*_obs_) were normalised to the total change in fluorescence at infinite concentration (Δ*F*_total_), giving rise to the ratio $\frac{{\Delta F_{obs} }}{{\Delta F_{total} }}$. The dissociation constant, *K*_d_, for TNP-ATP was calculated using Eq. (1):

1 $$ \frac{{\Updelta F_{obs} }}{{\Updelta F_{total} }} = \frac{{(K_{d} + L_{t} + E_{t} ) - \sqrt {K_{d} + L_{t} + E_{t} - 4L_{t} E_{t} } }}{{2E_{t} }}, $$

where *L*_t_ is the total ligand concentration (TNP-ATP) and *E*_t_ is the total protein concentration.

## Results

### Secondary structure prediction of the ATP-lid of CSK

**Fig. 1:**
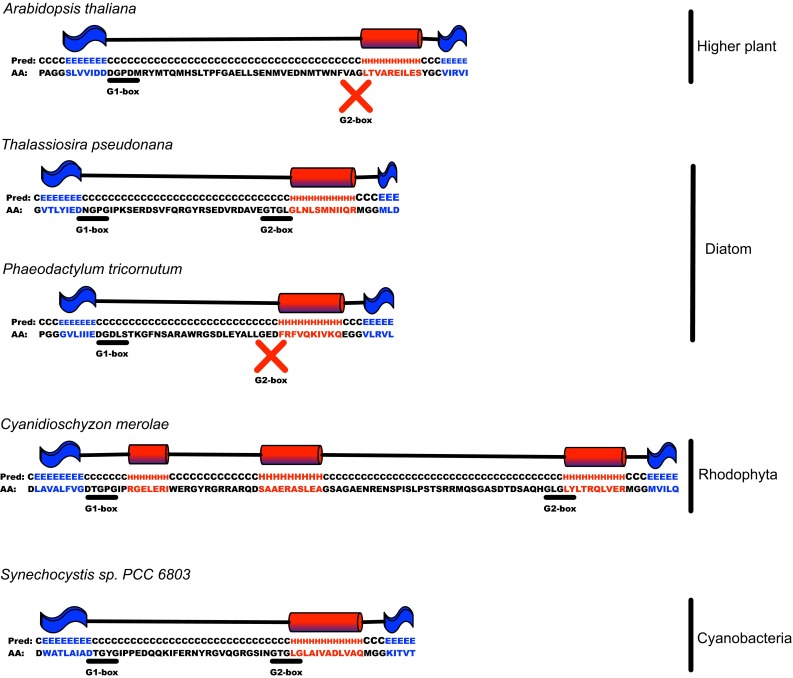
Predicted secondary structure of the ATP-lid of CSK homologues. Secondary structure of the ATP-lid of cyanobacterial Hik2 and of CSK of *Cyanidioschyzon merolae, Thalassiosira pseudonana, Phaeodactylum tricornutum, and Arabidopsis thaliana* predicted using the programme PSIPRED (http://bioinf.cs.ucl.ac.uk/psipred/). The conserved motifs G1 and G2 *boxes* are shown. Where the motif is missing, this is indicted by the *red cross*. AA stands for the target sequence and Pred for the predicated secondary structure. Coil is shown with *black line*, helix with *red oval* shape, and strand with *blue wave*

In order to gain insight into the catalytic domain of CSK, we employed secondary structure prediction. Figure 1 shows the predicted secondary structural elements of cyanobacterial and chloroplast CSKs. With the exception of *Cyanidioschyzon merolae* CSK, the cyanobacterial and chloroplast CSKs contain a similar secondary structure for the ATP-lid, which is located between the G1 and G2 motifs and is formed of an unstructured coil region. Interestingly, the *Cyanidioschyzon merolae* CSK contains an unusually large amino acid sequence between the G1 and G2 boxes, perhaps suggesting a larger ATP-lid.

### CSK contains a modified CA domain

**Fig. 2:**
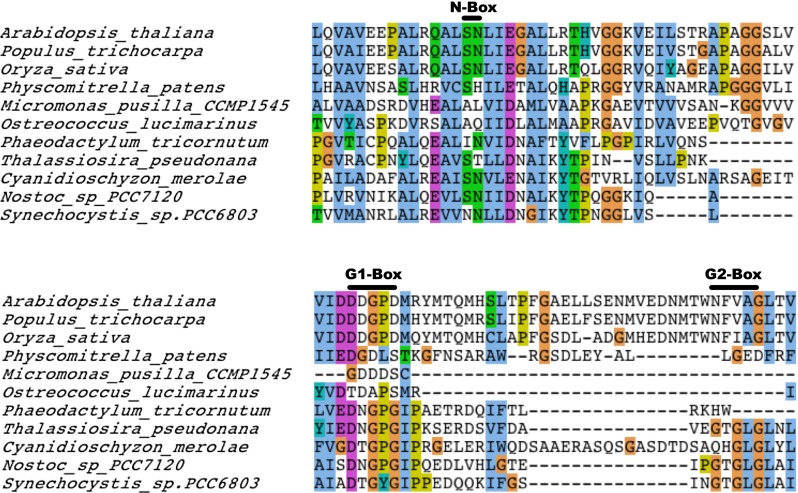
Conserved sequence features of CA domain of CSK. Sequence alignment of higher plant CSK and its bryophyte, algal, and cyanobacterial homologues. The residue *colours scheme* is the same as in Fig. 4. The N, G1, and G2 *boxes* are shown above the sequence

The ATP-binding cavity of histidine kinases contains conserved motifs that are essential for their histidine autophosphorylation. These include the G1 and G2 boxes, which have characteristic signatures of “DxGxG” and “GxGxG,” respectively. Sequence alignment of CSK homologues shows that plant CSKs have a modified ATP-binding domain (Fig. 2). In higher plants, only the first conserved glycine residue is retained and the second conserved glycine residue in the G1-box is replaced by an aspartic acid (Fig. 2). Moreover, the first two conserved glycine residues in their G2-box are replaced by an asparagine and a valine, respectively. Interestingly, they still retain the third glycine residue in their G2-box (Fig. 2). Some of the algal CSKs, likewise, do not possess a fully conserved G2-motif.

### Recombinant protein production

**Fig. 3:**
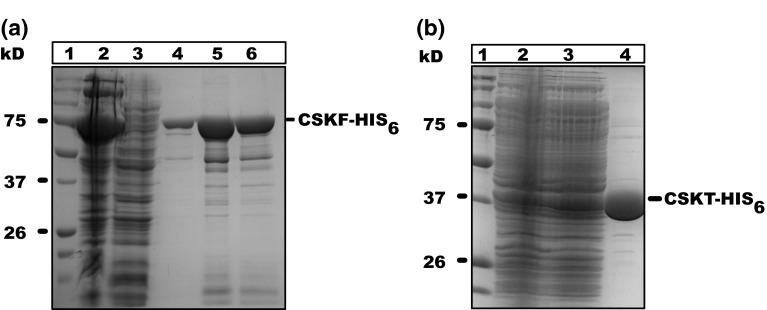
Protein overexpression and purification. The full-length CSK protein was overexpressed and purified as described in the experimental section. The following samples were loaded on a SDS-PAGE 12 %. *lane* 1, protein molecular weight marker; *lane* 2, total cell lysate; *lane* 3, soluble cell fraction; *lane* 4 and 5 are elution fraction1. On the left, protein molecular weight markers are shown in kDa. The positions of the overexpressed CSK_F and CSK_T are indicated on the *right*

We successfully cloned the *Arabidopsis* *CSK* gene and overexpressed and purified the recombinant protein from *E. coli*. Figure 3 shows overexpressed and purified full-length and truncated forms of *Arabidopsis* CSK. Figure 3a, lane 2 shows the full-length CSK protein being present mainly in the insoluble cell fraction and only in very low abundance in the soluble cell fraction (lane 3). The purified full-length CSK migrated on the SDS-PAGE with an apparent molecular mass of 75 kDa. The truncated form of CSK, containing the core kinase domain of CSK, migrates with an apparent molecular mass of 37 kDa.

### CSK does not autophosphorylate in vitro

**Fig. 4:**
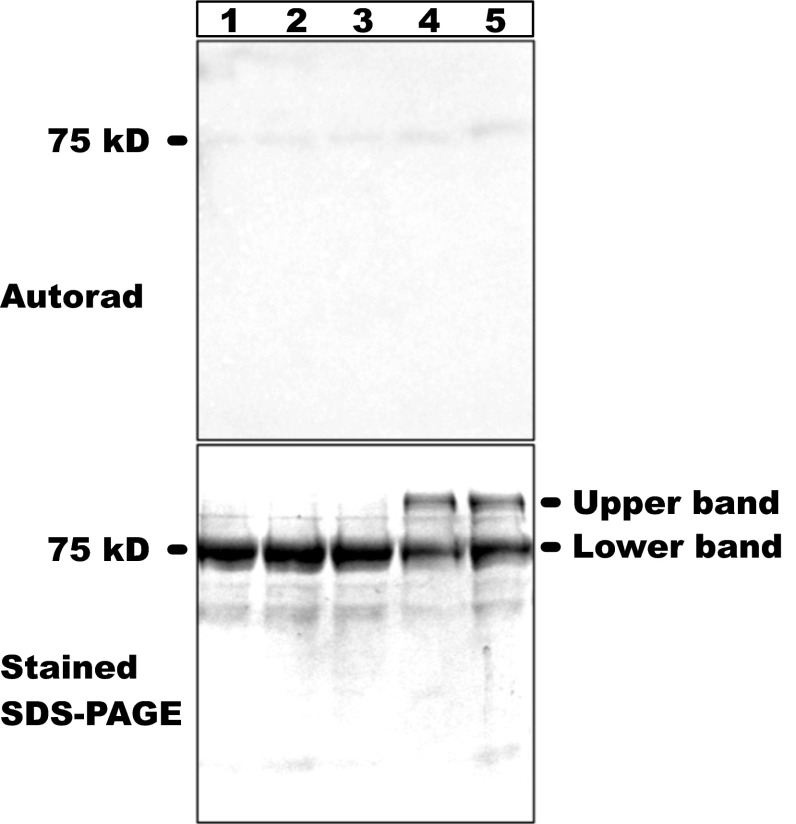
Effect of different redox agents on the activity of CSK. An autoradiograph of the reaction of CSK proteins pre-treated with *lane* 1, nothing; *lane 2*, 2 mm K_3_Fe(CN)_6_; *lane* 3, 6 mm DTT; *lane* 4, 0.5 mm benzoquinone; lane 5, 0.5 mm hydroquinone

Most protein kinases are autokinase active in that they phosphorylate their own amino acids before phosphorylating their substrates. We therefore examined autophosphorylation activity of full-length CSK in vitro in the presence of different redox agents, which might modulate its kinase activity. The autoradiograph in Fig. 4, lane 1, shows that untreated CSK is inactive as an autokinase. Treatment of CSK with different redox agents did not yield an autokinase active CSK (Fig. 4, lanes 2–5). Interestingly, benzoquinone and hydroquinone-treated CSK proteins migrated as two bands on a 12 % SDS-PAGE (Fig. 4, lane 4, 5).

### CSK binds an ATP analogue TNP-ATP

**Fig. 5:**
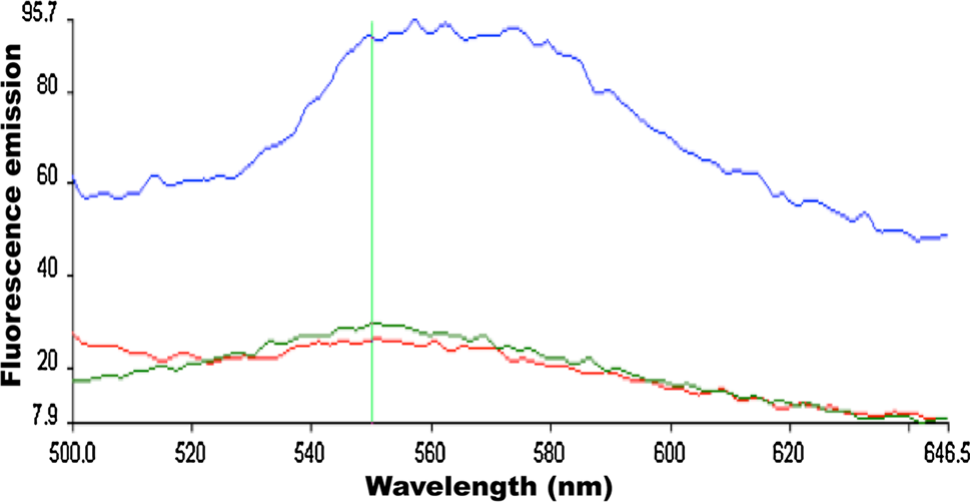
Binding equilibrium of TNP-ATP to *Arabidopsis* CSK using fluorescence spectroscopy. TNP-ATP was excited at 410 nm and excitation emission was measured between 500 and 646 nm. Fluorescence emission spectra of 1 µm TNP-ATP is shown by *red line*; fluorescence spectra of 1 µm TNP-ATP in the presence of 2 µm CSK is shown by *blue line*; and fluorescence spectra of 1 µm TNP-ATP in the presence of 2 µm CSK and 40 mm natural ATP is shown by *green line*

Based on sequence alignment shown in Fig. 2, CSK is predicted to contain an ATP-binding cavity, albeit with some modifications. However, the recombinant CSK protein seems to be incapable of an autophosphorylation reaction in vitro (Fig. 4). In order for CSK to function as a protein kinase, it must have a demonstrable ability to bind an ATP molecule and then catalyse substrate phosphorylation. Here, we investigated the ATP-binding activity of CSK using a fluorescent ATP derivative TNP-ATP. TNP-ATP contains a trinitrophenyl group at 2′(3′) hydroxyls of ATP. When exposed to a hydrophobic pocket such as an ATP-binding cavity, TNP-ATP becomes more fluorescent. Figure 5 shows the fluorescence emission spectrum of TNP-ATP in the presence of 2 µm CSK (Fig. 5, blue line). The fluorescence emission from TNP-ATP at 550 nm was increased by more than two-folds in the presence of CSK, indicating that TNP-ATP-CSK complex has been formed. The TNP-ATP from CSK can be displaced with the addition of an excess amount of natural ATP (Fig. 5, green line).

### TNP-ATP-binding constant

**Fig. 6:**
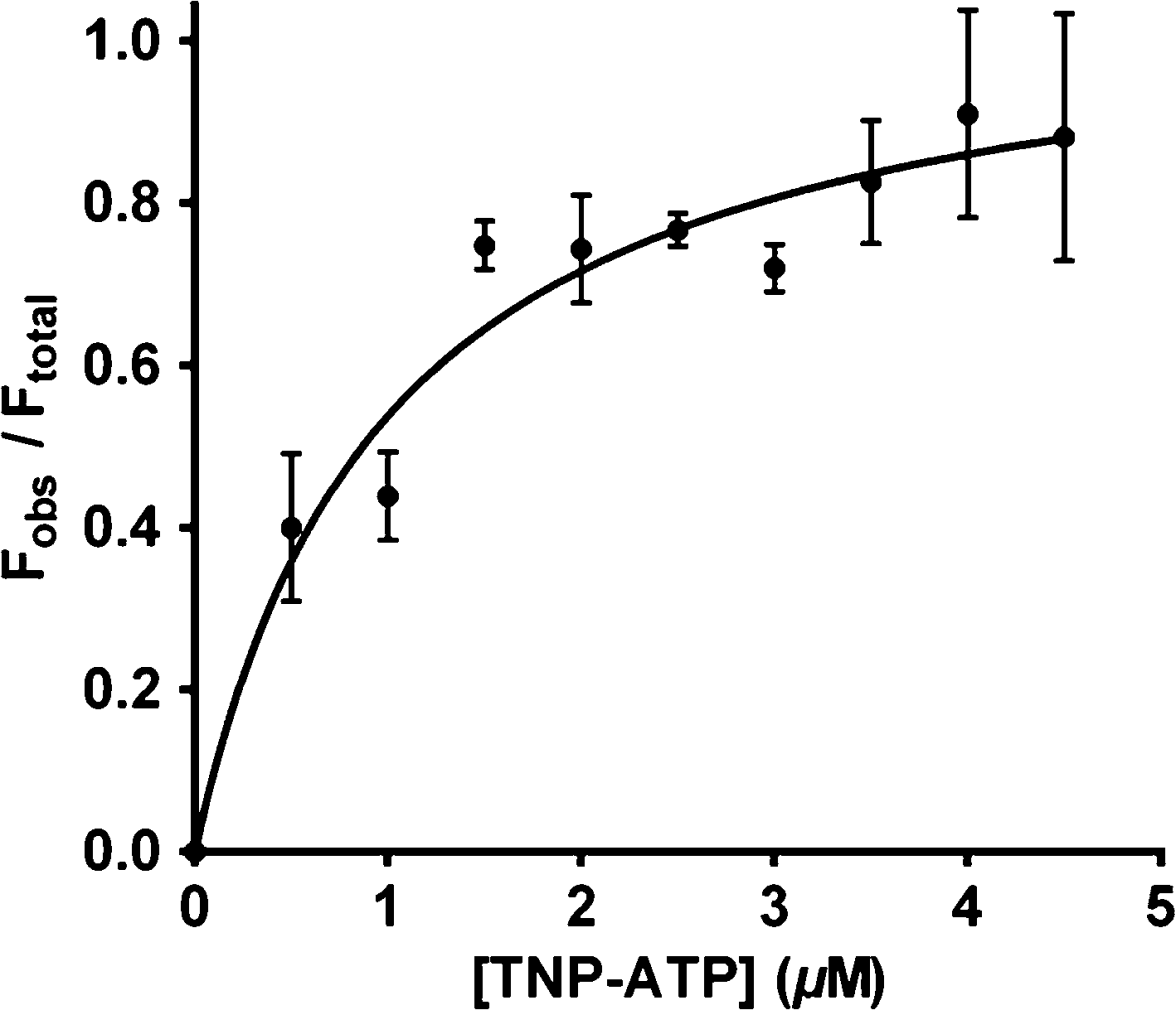
Titration of *Arabidopsis* CSK with TNP-ATP. TNP-ATP-binding titration was performed with varying concentration of TNP-ATP in the presence of 2 µm CSK and 1 µm ATP. TNP-ATP was excited at 410 nm and excitation emission at 550 nm was monitored. Correction to background TNP-ATP fluorescence was made by subtracting values for buffer plus TNP-ATP from CSK plus TNP-ATP and data were then plotted using prism 5 (Motulsky and Christopoulos 2003). Each data point represents the mean ±S.E of three measurements. Dissociation constant (*K*_d_) for TNP-ATP was calculated by nonlinear regression curve fitting of data

We next investigated CSK’s binding affinity for TNP-ATP by varying the concentration of TNP-ATP. The fluorescence emission increase at 550 nm was measured and data were fitted to a nonlinear regression model to calculate *K*_d_ for TNP-ATP. Figure 6 shows a *K*_d_ value of 1 µm.

### TNP-ATP dissociation constant

**Fig. 7:**
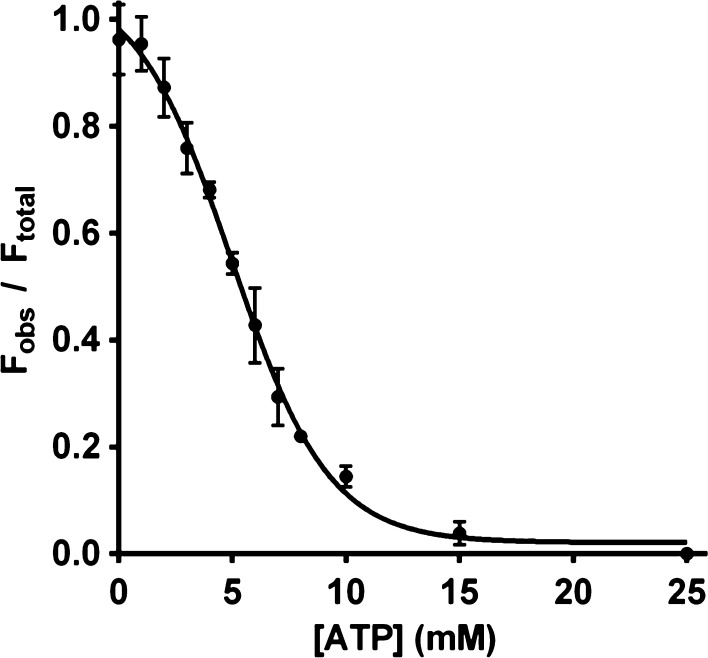
Displacement of TNP-ATP from *Arabidopsis* CSK. TNP-ATP displacement titration was performed with varying concentration of ATP to a sample that was pre-equilibrated with 1 µm TNP-ATP and 1 µm CSK. Decrease in intensity of emission at 550 nm was measured. Data were plotted as a function of ATP concentration and were plotted using prism 5 (Motulsky and Christopoulos 2003). Each data point represents the mean ±S.E of three measurements. The dissociation constant (*K*_d_) for TNP-ATP was calculated by nonlinear regression curve fitting of data

We further looked at the competitive exchange of TNP-ATP with natural ATP. The dissociation constant was determined by titrating CSK with ATP in the presence of 1 µm TNP-ATP. The resulting fluorescence decrease of TNP-ATP was measured. Figure 7 shows that a *K*_d_ value of 4.97 mm for ATP can be obtained by fitting the flourescence data using a nonlinear regression model.

## Discussion

A signal transduction cascade by a two-component system is initiated when the histidine kinase, upon sensing an environmental signal, undergoes autophosphorylation in an ATP-dependent manner (Stock et al. 2000). In this study, we provide evidence concerning the nucleotide-binding activity of *Arabidopsis* CSK. CSK in green algal and land plants has lost the conserved histidine residue required for its autophosphorylation and phosphotransfer activities. The conserved histidine residue in CSK has been replaced by a tyrosine or a glutamine in green algae and by a glutamate in higher plants (Puthiyaveetil and Allen 2009; Puthiyaveetil et al. 2008). Correspondingly, there are similar modifications in the CA domain of these modified CSKs (Fig. 1). These modifications, however, does not affect its ATP-binding activity (Figs. 5, 6, 7). The binding affinity of CSK for the fluorescent analogue of ATP (TNP-ATP) is 1 µm, a value that is comparable to that of bacterial histidine kinases. For TNP-ATP, the EnvZ and CheA kinases have a *K*_d_ value of 1.9 and 1.7 µm, respectively (Table 2) (Plesniak et al. 2002; Stewart et al. 1998). Furthermore, TNP-ATP can be displaced from CSK using unmodified ATP (Fig. 5), implying that TNP-ATP binding has the same characteristics as that of ATP.

**Table 2:** Comparison of the binding affinities of the substrates TNP-ATP and ATP to *Arabidopsis* *thaliana* CSK, CheA (Stewart et al. 1998) and *E. coli* EnvZ (Plesniak et al. 2002)

Sensor histidine kinase	Species	Binding affinity	
		TNP-ATP (µm)	ATP (mm)
CSK	*Arabidopsis thaliana*	1.0	4.97
CheA	*E.coli*	1.7	6.0
EnvZ	*E.coli*	1.9	6.2

The overall structure of the CA domain of the histidine kinase is formed of a *α*/*β* sandwich fold, composed of three helices and five stranded *β*-sheets, which is distinct from that of serine/threonine or tyrosine kinases. However, the histidine kinase fold bears strong similarity to the ATP-binding domain of DNA topoisomerase II (Roca and Wang 1992), DNA Gyrase B (Ali et al. 1995), the chaperone Hsp90 (Panaretou et al. 1998), and the DNA repair enzyme MutL (Ban et al. 1999). The CA domain is characteristic of its conserved sequence motifs: N, F, G1, G2, and G3 boxes that are important for stabilisation of an ATP molecule and for the hydrolysis of the ATP *γ*-phosphate. The N-box and G1-box are involved in the stabilisation of the adenine ring of ATP. In particular, a conserved aspartic residue found within the G1-box forms a hydrogen bond with the amino N6 of the adenine base (Marina et al. 2001; Trajtenberg et al. 2010). This interaction is crucial for conferring specificity to ATP and to prevent the binding of other nucleotides such as GTP. The CA domains discriminate nucleotides such as GTP based on the fact that they lack the amino group required for forming a hydrogen bond with the carboxyl group of the conserved aspartic acid within the G1-box. The F-box, which is the less conserved region of CA domain, and the G2-box together form a flexible loop that acts as the ATP-lid and controls the entry of the ATP-Mg^2+^ complex and the release of the ADP-Mg^2+^ complex. Conserved glycine residues in the G2-box facilitate the flexibility of the ATP-lid (Marina et al. 2001). The G1-box is fully conserved in all CSKs. The G2-box, however, is less conserved in chloroplast CSKs and unrecognisable in higher plants (Fig. 1). Nonetheless, this did not affect their ATP-binding activity. In contrast to higher plant CSK, substitution of any of the glycine residues in the G1 or G2 boxes of cyanobacterial CSK results in an inactive kinase (Ibrahim et al. 2016) suggesting that the modified CSKs contain a different catalytic fold.

CSK homologue in cyanobacteria is able to autophosphorylate in vitro and transfer phosphoryl groups to its response regulators Rre1 and RppA. Furthermore, its autokinase activity can be inhibited by sodium ions (Ibrahim et al. 2016). Although the modified CSK binds ATP in vitro (Fig. 5), we could not detect its autophosphorylation (Fig. 4). This could be because of a number of reasons. Firstly, plant CSK could be autokinase inactive at its ground state and thus a signal may be required to activate it. In our assay, this signal may have been absent. Secondly, the CSK might require a cofactor(s) such as FAD for its redox sensing activity and that the recombinant and purified protein used in this study could be an incomplete apoenzyme. A third possibility is that the bacterially expressed recombinant CSK is not folded correctly for the kinase reaction. CSK may well have lost its ability to autophosphorylate and might function in a manner similar to some of the modified bacterial histidine kinases such as DivL. In contrast to DivL, however, CSK exhibits an ATP-binding affinity similar to prototypical histidine kinases, while DivL binds ATP analogue TNP-ATP poorly, with a *K*_d_ value of 57 µm (Childers et al. 2014). The strong binding affinity of CSK for ATP thus indicates that it has retained its kinase activity.

The lack of a demonstrable autokinase activity in CSK also suggests that the modified CSKs have a catalytic mechanism that differs from that of their cyanobacterial homologues. Perhaps this modification is necessary for CSK to acquire a new substrate, as its cognate response regulators Ycf29 and Ycf27 are missing from chloroplasts in the green lineages (Puthiyaveetil and Allen 2009). Indeed, *Arabidopsis* CSK protein interacts with plastid transcription machineries, including sigma factor 1 (SIG1) (Puthiyaveetil et al. 2013, 2010), a transcriptional initiation factor that has a prokaryotic origin. SIG1 is required to initiate *psbA* and *psaAB* transcription (Shimizu et al. 2010).

Histidine kinases do not transfer the *γ*-phosphate group directly from an ATP molecule to their substrate. They instead use the high energy of the phosphoramide bond of the phospho-histidine to facilitate the transfer of the phosphate to an aspartate residue of the response regulator. Phospho-serine and -threonine are, however, thermodynamically more stable than phosphoramide or acyl-phosphate, they therefore cannot passively transfer phosphate groups. Indeed, several modified histidine kinases, such as ETR2 (Moussatche and Klee 2004), plant phytochromes (Fankhauser et al. 1999), α-ketoacid dehydrogenase kinase (Lasker et al. 2002), and pyruvate dehydrogenase kinase (PDK) (Thelen et al. 2000) have lost their ability to catalyse His–Asp phosphotransfer. They have instead acquired a catalytic mechanism that is similar to that of serine/threonine kinases in that they now phosphorylate their substrates on serine or threonine residues. It is likely that the modified CSKs in green lineage also acquired a serine/threonine-type catalytic mechanism similar to that of modified histidine kinases, a property that is yet to be demonstrated experimentally.

The PQ pool has a standard midpoint potential of +50 mV, *n* = 2 (Silverstein et al. 1993b) and an effective redox potential that changes with fluctuating light distribution between the photosystems. These fluctuations may affect a specific redox sensor. For example, the redox state of the PQ pool controls the light-dependent phosphorylation of chloroplast light-harvesting complex II (LHC II) (Allen 1992; Allen et al. 1981). The LHC II kinase (STN7/STT7) is responsible for phosphorylation of LHC II (Bellafiore et al. 2005; Depege et al. 2003; Rochaix 2007). LHC II kinase has a midpoint potential of +48 mV, which is similar to that of PQ pool midpoint potential (Silverstein et al. 1993a). Some quinone pool sensors, however, do not utilise its redox signal. RegB, for example, interacts with the reduced and oxidised forms of ubiquinone with similar affinities, and only the oxidised form of the ubiquinone seems to switch-off the autophosphorylation of RegB (Wu and Bauer 2010). The autophosphorylation of RegB is, in fact, regulated through an allosteric effect triggered by binding of ubiquinone/ubiquinol rather than oxidation by ubiquinone (Wu and Bauer 2010). The UV absorption spectra and the primary amino acid sequence of CSK (results not shown) did not reveal potential spectral signatures or binding motifs characteristic of redox sensitive cofactors such as heme or flavin, which suggests that CSK may employ a RegB-type quinone sensing mechanism rather than taking part in direct redox sensing. We also noted that benzoquinone-treated and hydroquinone-treated CSK protein migrated as two bands on the SDS-PAGE gel that is both denaturing and reducing (Fig. 4, lanes 4 and 5). Cysteine residue in enzymes are known to react with quinones to form quinone-cysteine adducts that cannot be broken with reducing agents, for example by DTT used in our sample buffer (Li et al. 2005). Cysteine residues in CSK could be forming a thioether-quinone adduct. This observation further strengthens the quinone-binding activity of CSK. However, it is not yet clear whether the adduct formation still occurs with the plastoquinone in the thylakoid membrane.

One question that is still unclear is how a water-soluble redox sensor such as CSK sense the quinone redox signal located within the thylakoid membrane? PQ is a mobile electron carrier that shuttles electrons between PS II and cytochrome *b*_6_ *f* in the thylakoid membrane. For membrane-anchored redox sensors, quinone appears to be an attractive candidate as the signalling molecule; however, for soluble redox sensors, it is a more challenging prospect. Nevertheless, there are several soluble proteins that are quinone pool sensors. The NifL sensor protein regulates the N_2_-fixation (*nif*) genes in *Azotobacter vinelandii* (Grabbe and Schmitz 2003). NifL is a flavoprotein that contains a redox active FAD cofactor that is reduced by the quinone pool. The CikA (circadian input kinase A) sensor protein from *Synechococcus elongatus* PCC 7942 is a soluble protein that senses the PQ pool unaided by a redox-active prosthetic group (Ivleva et al. 2006). In order for CSK to sense the PQ pool, it must have the ability to associate itself with the thylakoid membrane and it must possess a binding site for PQ. For CSK, the ability to associate with the thylakoid membrane may be imparted by the amphipathic helices in its GAF sensor domain. Can the GAF domain in CSK also act as a binding pocket for the reactive, hydrophobic PQ head group? GAF domains are known for their ability to bind small cofactors such as heme and nucleotides (Sardiwal et al. 2005). CSK, indeed, binds DBMIB with a *K*_d_ value of 3.66 µm (Puthiyaveetil et al. 2013), a binding affinity comparable to other quinone binding proteins and thus strengthening the quinone-sensing activity of CSK.

Based on results presented here, we conclude that the catalytic domain of CSK became modified in evolution in order to accommodate a Ser/Thr kinases-type catalytic mechanism that was essential for its incorporation into an existing signal transduction network. CSK’s ATP-binding activity and ability to form quinone adducts further strengthen its role in the crucial sensory circuit that connects the redox state of the PQ pool with the chloroplast gene transcription, consistent with the CoRR hypothesis for the evolutionary retention of organellar genomes (Allen 1993a, b, 2003, 2015).
